# Endoscopic evaluation of middle ear anatomic variations in autopsy series: analyses of 204 ears^☆^

**DOI:** 10.1016/j.bjorl.2018.10.002

**Published:** 2018-11-03

**Authors:** Bayram Şahin, Kadir Serkan Orhan, Hızır Aslıyüksek, Erdoğan Kara, Yalçın Büyük, Yahya Güldiken

**Affiliations:** a Kocaeli Health Sciences University, Derince Training and Research Hospital, Department of Otorhinolaryngology – Head and Neck Surgery, Kocaeli, Turkey; b University of Istanbul, Istanbul Medical Faculty, Department of Otorhinolaryngology – Head and Neck Surgery, Istanbul, Turkey; c Ministry of Justice, Council of Forensic Medicine, Istanbul, Turkey

**Keywords:** Middle ear anatomy, Endoscopic ear surgery, Retrotympanum, Ponticulus, Subiculum, Anatomia da orelha média, Cirurgia endoscópica da orelha, Retrotímpano, Pontículo, Subículo

## Abstract

**Introduction:**

Microsurgery of the ear requires complete evaluation of middle ear surgical anatomy, especially the posterior tympanic cavity anatomy. Preoperative assessment of the middle ear cavity is limited by the permeability of eardrum and temporal bone density. Therefore, middle ear exploration is an extremely useful method to identify structural abnormalities and anatomical variations.

**Objective:**

The aim of this study is to determine anatomic variations of the middle ear in an autopsy series.

**Methods:**

All evaluations were performed in the Forensic Medicine Institute Morgue Department. The cases over 18 years of age, with no temporal bone trauma and history of otologic surgery included in this study.

**Results:**

One hundred and two cadavers were included in the study. The mean age was 49.08 ± 17.76 years. Anterior wall prominence of the external auditory canal was present in 27 of all cadavers (26.4%). The tympanic membrane was normal in 192 ears (94%) while several eardrum pathologies were detected in 12 ears (6%). Agenesis of the pyramidal eminence and stapedial tendon was found in 3 ears. While the ponticulus was bony ridge-shaped in 156 of 204 ears (76.4%), it was bridge-shaped in 25 ears (12.3%). The ponticulus was absent in 23 ears (11.3%). While complete subiculum was present in 136 of all ears (66.7%), incomplete subiculum was present in 21 ears (10.3%). Subiculum was absent in 47 ears (23%). Facial dehiscence was found in 32 ears and the round window niche was covered by a pseudomembrane in 85 ears (41.6%). A fixed footplate was present in 7.4% of all ears, and no persistent stapedial artery was seen in any cases.

**Conclusion:**

The pseudomembrane frequency covering the round window niche was found different from reports in the literature. In addition, the frequency of the external auditory canal wall prominence has been reported for the first time.

## Resumo

**Introdução:**

A otomicrocirurgia requer avaliação completa da anatomia cirúrgica da orelha média, especialmente da anatomia da cavidade timpânica posterior. A avaliação pré-operatória da cavidade timpânica é limitada pela permeabilidade do tímpano e densidade do osso temporal. Portanto, a exploração da orelha média é um método extremamente útil para identificar anormalidades estruturais e variações anatômicas.

**Objetivo:**

Determinar as variações anatômicas da orelha média em uma série de autópsias.

**Método:**

Todas as avaliações foram realizadas no necrotério do Instituto Médico-Legal. Os casos com mais de 18 anos, sem trauma do osso temporal e história de cirurgia otológica foram incluídos neste estudo.

**Resultados:**

Cento e dois cadáveres foram incluídos no estudo. A média de idade foi de 49,08 ± 17,76 anos. A proeminência da parede anterior do conduto auditivo externo estava presente em 27 de todos os cadáveres (26,4%). A membrana timpânica era normal em 192 orelhas (94%), enquanto várias alterações do tímpano foram detectadas em 12 orelhas (6%). Agenesia da eminência piramidal e do tendão do estapédio foi encontrada em 3 orelhas. Enquanto o pontículo tinha formato de crista óssea em 156 das 204 orelhas (76,4%), tinha o formato de ponte em 25 orelhas (12,3%). O pontículo estava ausente em 23 orelhas (11,3%). Enquanto o subículo completo estava presente em 136 de todas as orelhas (66,7%), encontrava-se incompleto em 21 orelhas (10,3%). O subículo estava ausente em 47 orelhas (23%). Deiscência facial foi encontrada em 32 orelhas e o nicho da janela redonda estava coberto por uma pseudomembrana em 85 orelhas (41,6%). A platina fixa foi observada em 7,4% de todas as orelhas e a artéria estapediana persistente não foi vista.

**Conclusão:**

A frequência da pseudomembrana que cobre o nicho da janela redonda foi diferente daquela encontrada na literatura. Além disso, a frequência da proeminência da parede do canal auditivo externo foi relatada pela primeira vez.

## Introduction

Modern otologic microsurgery requires complete evaluation of middle ear surgical anatomy, especially the posterior tympanic cavity anatomy, because this region contains many irregular spaces, thus creating a suitable environment for hiding of cholesteatoma.^1^, ^2^ The posterior tympanic cavity contains four sinuses that surround the facial nerve and the Fallopian canal. There are two sinuses located on lateral side of the Fallopian canal: the facial sinus, which is in the superior, and lateral tympanic sinus, which is in the inferior, and they are separated from each other by the chordal notch. The other two sinuses are in the middle of the Fallopian canal: posterior tympanic sinus, which is in the superior, and sinus tympani, which is in the inferior, and they are separated from each other by the ponticulus.^2^, ^3^ The sinus tympani is the posterior extension of the mesotympanic space towards to the tympanic annulus. The superior border is constituted by ponticulus while inferior border is formed by subiculum.^4^

In recent years, increased use of endoscopes in middle ear surgery has provided hidden details of middle ear anatomy.^2^, ^5^, ^6^, ^7^, ^8^, ^9^, ^10^, ^11^ It is almost impossible to explore the sinus tympani, anterior epitympanic and retrotympanic space by a conventional microscopic approach. Endoscopes provide a clearer and wider view of middle ear anatomy and, they also help in a better understanding of middle ear physiology and pneumatization patterns.

Preoperative evaluation of the middle ear space is limited by the permeability of tympanic membrane (TM) and temporal bone density.^12^ It is possible to determine the structure of the middle ear ossicles, pneumatization of middle ear and mastoid bone, and various bone pathologies by evaluation of the temporal bone with high resolution computerized tomography. However, middle ear exploration is required to make more detailed evaluations and to identify structural abnormalities because of the small size of middle ear structures and complex anatomy.

In this study, we aimed to determine the middle ear anatomic variations with endoscopic evaluation performed in fresh cadavers, and to compare the obtained results with the current medical literature.

## Material and methods

One hundred and two cadavers (a total of 204 ears), which were sent to Institute of Forensic Medicine for autopsy, were evaluated between June and December 2017. The cases over eighteen years of age with no temporal bone fracture, head trauma or history of otologic surgery were included in this study. An institutional board review approved the study. The ethical approval was granted by the Scientific Board of the Forensic Medicine Institute (Project no. 21589509/2017, 23/05/2017). All evaluations were performed in the Morgue Department within the guidelines of a standard autopsy procedure.

Cadavers with previous otologic surgery findings and congenital external auditory canal and/or auricular atresia were excluded from the study. The cadavers whose causes of death were gunshot injury, penetrating trauma, drowning, freezing and burning, were also excluded.

### Surgical procedure

All otologic dissections and anatomic evaluations were performed with a transcanal approach using the 0° and 45° endoscopes and a portable endoscopy unit (4 mm Hopkins telescope, Karl Storz, Tuttlingen, Germany). Initially, the structure of the external ear canal, the anterior wall prominence (AWP) and presence of bony dehiscence in the anterior wall were evaluated. AWP was classified according to what percentage of the TM can be seen when it is viewed from the cartilage/bone junction of the external ear canal with a 0° endoscope. It was classified as follows: Type 1 (more than 75%), Type 2 (50%–75%) and Type 3 (less than 50%). Findings such as perforation, myringosclerosis, retraction pocket, pseudomembrane, and cholesteatoma present in the TM were recorded. The Rosen incision was performed at a distance of 5 mm from the tympanic annulus and a tympanomeatal flap was elevated. After that middle ear cavity was fully visualized.

The presence of pathologies such as middle ear effusion, hemotympanum, tympanosclerosis and cholesteatoma was assessed. Decreased movement and loss of integrity of the ossicular chain and stapes footplate fixation were noted. Stapes footplate movement was evaluated before the incudostapedial joint was removed with a curved pick, and incus and malleus were removed without any damage to the structural integrity.

At this stage, persistent stapedial artery, presence of protrusion towards the stapes footplate on the tympanic segment of facial nerve canal, and what percentage of the stapes footplate can be seen was noted. Subsequently, a 90° curved pick was placed under the superstructure and stapes separated from the oval window (OW) as in one piece with footplate.

Facial nerve canal dehiscence (FNCD), ponticulus, subiculum, presence of pseudomembrane on the round window (RW) niches, and position of the RW according to the OW were examined (the position of the OW was accepted as the 12 o’clock position and the position of the RW was determined according to the clockface).

**Figure 1:**
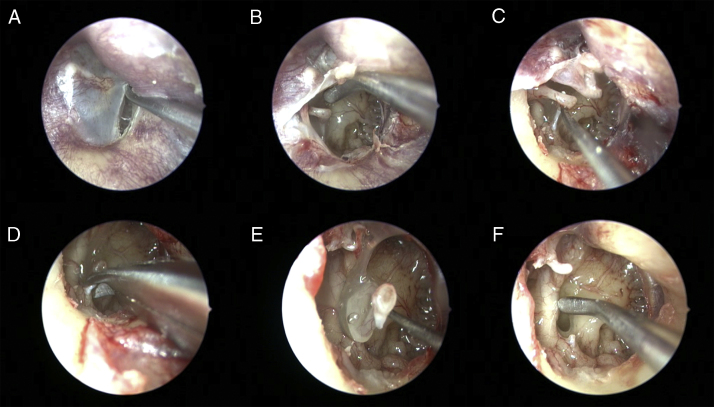
Right ear. (A) Elevation of the tympanomeatal flap from external auditory canal. (B) Visualization of the middle ear cavity via transcanal endoscopic approach. (C) Disconnection of the incudostapedial joint by 90° curved pick. (D) Removing of the stapedial tendon by curved micro-scissors. (E) Separation of the stapes from the oval window as in one piece with footplate. (F) Evaluation of the facial nerve canal dehiscence by micro elevator.

The presence of high jugular bulb (HJB), dehiscence jugular bulb (DJB), internal carotid artery (ICA) dehiscence and Jacobson nerve were examined. Following completion of all evaluations, the tympanomeatal flap was replaced into anatomic position and dissection was terminated (Fig. 1).

## Results

One hundred and two cadavers (81 male and 21 female) were included into the study. The mean age was 49.08 ± 17.76 years (min. 18 years, max. 86 years, 48.23 ± 16.84 years for males and 52.38 ± 21.05 years for females).

**Figure 2:**
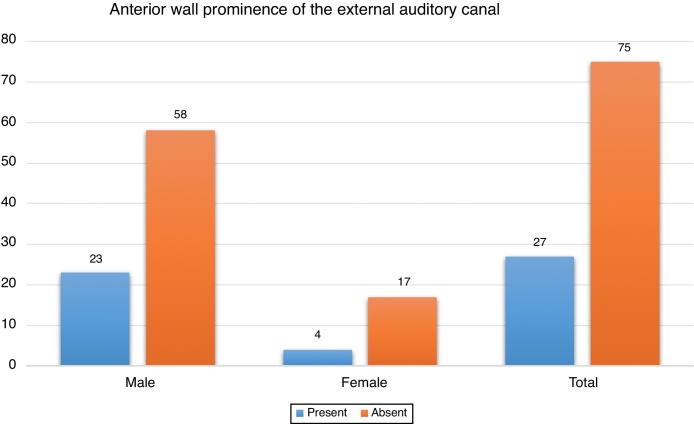
Distribution of anterior wall prominence of the external auditory canal according to gender.

Twenty-seven cadavers (54 ears, 26.4%) had AWP of the external auditory canal. Of these ears, 33 (66.1%) were Type 1 (more than 75% of TM was visible), 15 (27.8%) were Type 2 (50%?75% of TM was visible) and remaining 6 (11.1%) were Type 3 (less than 50% of TM was visible). When both genders were evaluated separately, the incidence in males was 28.3% (23/81), while the frequency in females was 19% (4/21) (Fig. 2). In all cases, the AWP of the external auditory canal was seen bilaterally. In none of the cases was bony dehiscence detected in the anterior wall of the external auditory canal.

While the structure and integrity of the TM was normal in 192 ears (94%), various eardrum pathologies were detected in 12 ears (6%). Of these ears, four had an old perforation area closed with pseudomembrane, three had a central perforation, two had adhesive otitis media (Sade Type 4) with cholesteatoma, two had an atrophic membrane and one had an epitympanic perforation with cholesteatoma. In addition, myringosclerosis was found in 19 ears (9.3%) without perforation.

The integrity of middle ear ossicles was normal in 200 ears (98%) while various ossicular chain pathologies were detected in 4 ears (2%). While erosion of the long arm of the incus was present in 3 ears, erosion of the malleus head, long arm of incus and stapes superstructure were detected in remaining one ear. We observed TM retraction in all ears which had an ossicular chain defect and there was cholesteatoma in three of them. In 23 ears (11.3%), although middle ear ossicle integrity was complete, there was a decrease in mobility of the ossicular chain. Of these ears, 16 (16/204, 7.84%) had fixation at the base of the stapes while the stapes movement was found normal in the remaining 7. In 31 (15%) of all ears, mucosal adhesions were found around the stapes footplate. However, when 6 ears with fixation in the stapes footplate were excluded, mucosal adhesions were associated with a decrease in ossicular chain movement in only 5 ears. Although mucosal adhesions were found around the middle ear ossicles in 20 of these 31 ears, ossicular chain movement was completely normal.

**Figure 3:**
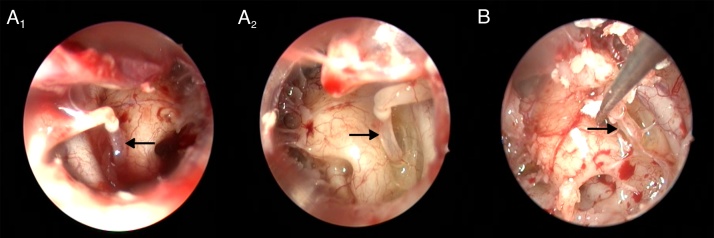
Agenesis of the pyramidal eminence and stapedius tendon. (A_1_) Case I right ear; (A_2_) Case I left ear; (B) Case II left ear (Black arrows: stapes).

The pyramidal eminence, stapedius muscle and tendon agenesis were found in 3 ears, which was observed bilaterally in one cadaver and unilateral (left) in another one (Fig. 3). In both cases, ossicular chain integrity and movement were normal. No other abnormality was found in their medical records and autopsies. In all other ears (201 ears, 98.5%) the pyramidal eminence was funnel shaped and the tendon was held in the stapes superstructure.

**Figure 4:**
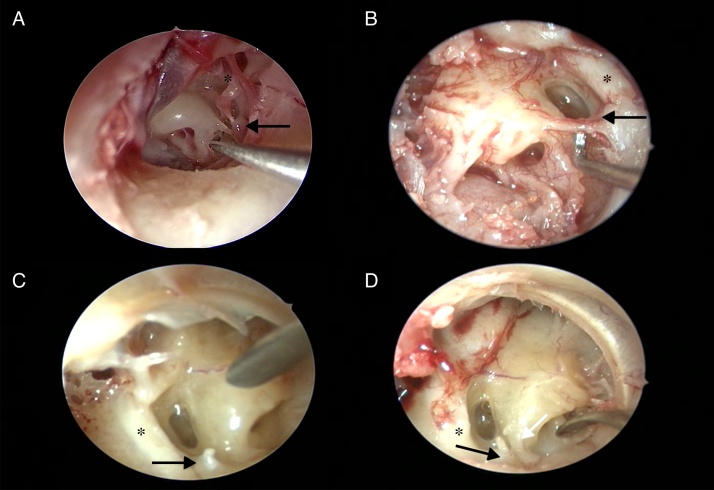
Variations of the ponticulus. (A) Bridge shape (indicated by 90° curved pick). (B) Bony ridge shape (indicated by 90° curved pick). (C) Total absence. (D) Total absence of ponticulus and bony ridge shape subiculum (indicated by white arrow) (black arrow, pyramidal eminence; *, facial nerve).

The ponticulus was observed as a ridge-shaped in 156 ears (76.4%), while it was found bridge-shaped in 25 ears (12.3%). In the presence of a bridge-shaped ponticulus, it was seen that the posterior tympanic sinus and the sinus tympani were connected to the inferior of the ponticulus. The ponticulus was absent in 23 ears (11.3%) and, sinus tympani and posterior tympanic sinus widely linked to each other in all of these cases (Fig. 4 and Table 1).

**Table 1:** The comparison of ponticulus shapes and their frequency in different studies.

	Number of ears	Incidence of ponticulus	Bony ridge	Bridge shape	Incomplet	Absent
Holt^17^	50 temporal bones	80% (40/50)	NA	NA	7/50	10/50
Cheiţă et al.^19^	37 temporal bones	83.8% (28/37)	16/37	12/37	3/37	6/37
Bonali et al.^20^	42 patients and 83 cadavers	100%	47/125	44/125	34/125	NA
Marchioni et al.^13^	38 ears in clinical study	89.5% (34/38)	32/38	2/38	4/38	NA
Our study	204 ears in cadavers	88.7% (181/204)	156/204	25/204	NA	23/204

**Figure 5:**
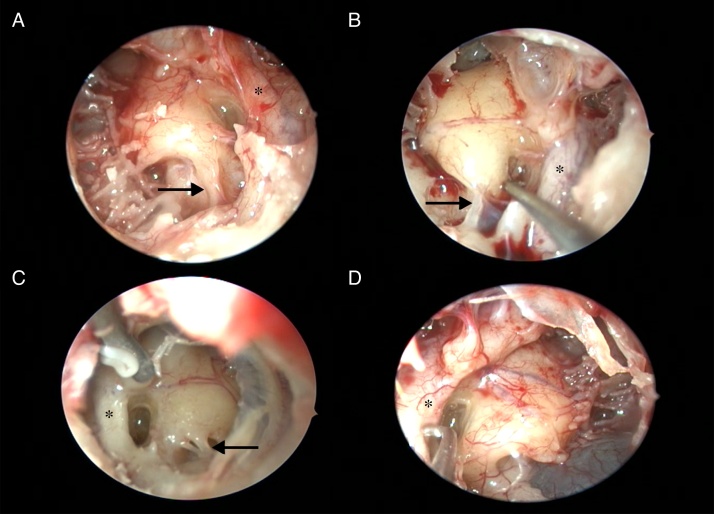
Variations of the subiculum. (A) Bony ridge shape. (B and C) Bridge shape. (D) Total absence and dehiscence jugular bulb abnormality (black arrows: subiculum; *, facial nerve).

Complete subiculum was detected in 136 ears (66.7%), while incomplete subiculum was found in 21 ears (10.3%). Of these years, 132 had ridge-shaped subiculum and 4 had bridge-shaped. In the presence of a bridge-shaped subiculum, it was seen that the sinus tympani and hypotympanic air cells were connected to inferior of the subiculum. In the 47 ears (23%), no subiculum was present and it was observed that the sinus tympani, hypotympanic cells and RW niches were linked to each other (Fig. 5).

FNCD of the tympanic segment was observed in 32 ears (15.7%). Protrusion of the facial nerve canal was also found in 9 ears (4.4%), extending towards the stapes footplate and preventing the OW being fully visible. In 6 of these 9 ears ¾ of the OW, in 2 ears ½ of the OW and in one ear ¼ of the OW could be visualized. In 6 of these 9 ears, a bony dehiscence was detected in the facial nerve canal simultaneously.

The RW niche was covered by a pseudomembrane in 85 ears (41.6%). Of these ears, 47 (23%) had complete and another 38 (18.6%) had incomplete membrane. When the position of the RW according to the OW was evaluated (the OW is accepted as 12 o’clock position), it was seen at 7 o’clock position in right ear and the 5 o’clock position in left ear. The Jacobson nerve was clearly identified on the promontorium in all of the cases. HJB was detected in 17 ears (8.3%), and DJB was present 3 ear (1.47%), and ICA dehiscence was found in only 2 ears (0.9%). The anterior tympanic fold was covered by mucosal membrane in 41 ears (20.1%) whereas the posterior tympanic fold was enclosed only in 4 ears (1.9%) (Table 2).

**Table 2:** Incidence and clinical importance of both external and middle ear anatomical variations.

Anatomical Variation	Clinical importance	*n*
*External ear*		
Anterior wall prominence of the external auditory canal	May prevents to see the surgical field at various degrees during transcanal middle ear surgery	54 (26.4%)

*Middle ear (mesotympanum)*		
CT was located outside the bony canal	CT injury during surgery	17 (8.3%)

*Middle ear (retrotympanum)*		
Facial nerve canal dehiscence	Facial nerve injury during surgery	32 (15.6%)
Facial canal protrusion	Facial nerve injury during surgery and/or covering the stapes footplate	9 (4.4%)
Agenesis of the PE and stapedial tendon	Hyperacusis?	3 (1.4%)
Bridge shaped ponticulus	Residual cholesteatoma	25 (12.2%)
Bridge shaped subiculum	Residual cholesteatoma	4 (1.9%)
Pseudomembrane presence at the RW niche	Leads to reduction in the diffusion of drugs applied in the middle ear to the inner ear	85 (41.6%)

*Middle ear (hypotympanum)*		
High jugular bulb	Jugular bulb injury and bleeding	17 (8.3%)
Dehiscence jugular bulb	Jugular bulb injury and bleeding	3 (1.4%)
Internal carotid artery dehiscence	Internal carotid artery injury and catastrophic bleeding	2 (0.9%)

CT, corda tympani nerve; PE, pyramidal eminence; RW, round window.

## Discussion

Our study provides detailed information about middle ear anatomy, including variations as well as pathologies. These findings will be extremely useful in TM interventions and middle ear surgery.

The AWP of the external auditory canal may prevent surgeons from obtaining a complete surgical field at various degrees during transcanal middle ear surgeries. Although this situation is well known by otologists, there is no study in the literature that classifies this variation and reports its frequency. However, Marchioni et al.^13^ reported that external auditory meatus measurements did not impose any limitations during the surgical procedures of the 40 patients who were operated with transcanal endoscopic approach. The standard otomicroscopic surgical procedures do not provide enough angulation to visualize all parts of the TM, especially in patients with anterior TM perforation; however, by using angled endoscopes (30° and 45°) the TM and protympanum can be seen completely. It should be kept in mind that the anterior part of the eardrum cannot be seen when viewed with the surgical microscope during operation if the AWP of the external ear canal is present.

The surgical anatomy of the middle ear has been increasingly investigated in recent years with endoscopic studies. These studies usually focus on a specific anatomical region or formation; sinus tympani,^2^, ^13^, ^14^ inferior retrotympanum,^15^ RW region,^16^ ponticulus^17^ and subpyramidal space.^18^ Some of the bone protrusions that separate these anatomical formations may be in the form of a bridge, so that the regions may show partial or complete continuity with each other. This anatomic feature mentioned has a critical clinical importance in the presence of pathologies such as cholesteatoma. The cholesteatoma matrix may be hidden under these bridges-like structures, and that can result in residual disease or spreading of the disease easily from one region to another without bone erosion. For these reasons, if the hypotympanum and retrotympanum are affected by disease, a wide surgical exposure must be achieved using angled endoscopes.

Holt^17^ described the ponticulus as a bone notch extending from the pyramidal eminence to the promontory in a dissection study and reported its incidence as 80% (33 complete and 7 incomplete). Cheiţă et al.^19^ found the incidence of the ponticulus as 83.78% (31/37) in the temporal bone dissection study. Of these 31 temporal bones, 16 had bony ridge-shaped, 12 had bridge-shaped ponticulus, whereas 3 has incomplete ponticulus. Bonali et al.^20^ reported incidence of complete ponticulus as 73% (bony ridge 38% and bridge shape 35%) in the series which includes 42 patients and 83 cadavers. In this study, the incidence of incomplete ponticulus was reported as 27%, while the complete absence of ponticulus was not detected in any case. Marchioni et al.^13^ reported the incidence of ponticulus as 89.5% (34/38) in their series. In our study, the ponticulus was found as a bony ridge in 84.2% of the patients, whereas a bridge-shaped ponticulus was detected in 5.3% of the patients, and an incomplete ponticulus was present in 10.5% of all patients. Additionally, the incidence of ponticulus was detected as 88.7% (181/204).

Cheiţă et al.^19^ reported that the rate of complete subiculum absence as 24.32% (9/37) in their study. Bonali et al.^20^ reported this rate as 34% (42/125), whereas Măru^21^ described as 18% (9/50). Marchioni et al.^18^ found the ratio of subiculum absence as 16% in the series of 25 patients who had middle ear cholesteatoma. In our study, complete subiculum absence was 23% (47/204) similar to the results of Cheiţă et al.^19^ These variabilities in the incidence of subiculum and ponticulus may be related to differences in patient numbers, racial traits, and increased frequency of variations in this anatomic structure.

Di Martino et al.^22^ evaluated 357 patients who underwent middle ear surgery and 300 temporal bone specimens in terms of FNCD. They found that the presence of intraoperative FNCD was most common around the OW (16/23) in their study, and its frequency reported as 6.4% (23/375). In the other part of this study, which includes autopsy series, they found FNCD frequency as 19.7% (59/300). Boroń et al.^23^ reported the frequency of FNCD as 15.5% (7/45) in their series of 45 patients who were treated for a middle fossa or posterior fossa bony defect. We found the frequency of FNCD as 15.7% (32/204) in our autopsy series. In our study, we found that FNCD was most commonly located around the OW similarly with the literature. The greater frequency of FNCD seen in autopsy series or in temporal bone dissection studies relative to clinical trials may be related to the better visualization of the tympanic segment of the facial nerve canal during the autopsy. If the tympanic segment of the nerve is assessed from different angles (via 30° and 45° endoscope) and better magnification by endoscope (compared with the microscope), even the smallest dehiscence can be detected.

We found agenesis of both the stapedial tendon and the pyramidal eminence in 3 of all ears (1.47%). The TM, ossicular chain integrity, position and movement were normal in these two cadavers, and there were neither congenital nor acquired disease present to explain this abnormality. The agenesis of the stapedial tendon and pyramidal eminence is an extremely rare congenital malformation of the middle ear. There are only few reports have been described by several authors in the literature.^24^, ^25^, ^26^

The RW chamber is a three-dimensional space extending between the RW niche and the RW membrane. The RW niche, which is especially important in cochlear implant and cholesteatoma surgeries, is sometimes covered by a pseudomembrane. Marchioni et al.^16^ reported the incidence of pseudomembrane in the RW niche as 9.5% (4/42) in their study. Unlike Marchioni et al.,^16^ the incidence of that variation was found as 41.6% (85/204) in our series. This wide variation between the two studies may be related to the high number of patients in our series and to racial traits. It is known that pseudomembrane may form in the RW niche due to previous middle ear infections. However, since this information was not available in the medical records of the cadavers involved in the study, the relationship between infections and pseudomembrane development could not be demonstrated. This anatomic variation may be significant in terms of influencing the success of intratympanic injection therapy.

Although vascular malformations of the temporal bone are rarely seen, they are extremely important in middle ear surgery. These vascular anomalies include; ICA dehiscence, aberrant ICA, HJB, DJB and persistent stapedial artery.^27^, ^28^ The rarest vascular malformation of the temporal bone is the presence of aberrant ICA and its incidence was reported to be less than 1%.^29^, ^30^ The clinical signs and symptoms of this abnormality are not specific, but the most common symptom is hearing loss. Symptoms such as pulsatile tinnitus, ear infections and ear pain can also be seen.^29^, ^30^, ^31^ During the otoscopic examination, it can be observed as a mass with reddish color on the anterior-inferior part of the TM. Despite, the fact that we have not encountered the presence of aberrant ICA in our study, bony dehiscence of ICA was detected in 2 cadavers (0.9%).

Different descriptions of HJB are available in the literature: (1) The jugular bulbus apex should be above the superior tympanic annulus, or basal turn of cochlea and RW level; (2) It should be on the inferior wall of the external auditory canal; (3) The distance between the jugular bulb and the ICA inferior wall should be shorter than 2 mm; (4) It should be above the level of the cochlear aqueduct; (5) It should be closely related to the ICA and endolymphatic duct in medial position, or that ought to protrude in the hypotympanum or mesotympanum.^32^, ^33^, ^34^, ^35^, ^36^, ^37^ The incidence of HJB is reported as 6%?20% in radiological study,^33^ while it is indicated as 3%?65% in clinical and anatomical studies.^28^, ^34^, ^36^, ^37^

Atmaca et al.^38^ reported the incidence of HJB frequency as 15.3% and DJB frequency as 7.5% in their radiological study. Sayit et al.^39^ described the incidence of the HJB frequency as 22% and DJB frequency as 3.4% in the radiological study which includes 3285 patients. However, there are different studies in the literature reporting that the incidence of HJB and DJB changes between as 3.5%?22.6% and 0.5%?1.7% respectively.^40^, ^41^ In our study, we found that HJB frequency as 8.3% (17/204) and DJB frequency as 1.47% (3/204), similar to the literature. The higher incidence of HJB in radiological studies compared to anatomical studies may be related to the possibility of evaluating a wider range from different angles with radiological imaging methods, and differences in the definition of this variation.

## Conclusion

It is of great importance to know the anatomical variations of the external ear canal and middle ear in preoperative planning for otological surgeries. In our study, findings such as FNCD, HJB, DHB, ICA dehiscence, ponticulus and subiculum incidence were found to be consistent with the literature, but the pseudomembrane frequency covering the RW niche was found to be higher than that cited in the literature. In addition, the frequency of the external auditory canal wall prominence has been reported for the first time.

Our study includes a sufficient number of patients when compared with similar studies. However, there is a need for larger series to be made with higher numbers and different races.

## Ethical approval

The ethical approval was granted by the Scientific Board of the Forensic Medicine Institute (*Project number: 21589509/2017, 23/05/2017*).

## Conflicts of interest

The authors declare no conflicts of interest.
